# Supplementary material for: VCF1 is a p97/VCP cofactor promoting recognition of ubiquitylated p97-UFD1-NPL4 substrates
Source: Nat Commun. 2024 Mar 19;15:2459. doi: 10.1038/s41467-024-46760-4 (PMC10950897; doi:10.1038/s41467-024-46760-4)
Supplement: Supplementary file 1 — Supplementary Information [file 41467_2024_46760_MOESM1_ESM.pdf]

Supplementary Information

VCF1 is a p97/VCP cofactor promoting recognition of ubiquitylated p97-UFD1-NPL4 substrates

Ann Schirin Mirsanaye[1,*], Saskia Hoffmann[1,*], Melanie Weisser[1], Andreas Mund[1], Blanca Lopez Mendez[1], Dimitris Typas[1], Johannes van den Boom[2], Bente Benedict[1], Ivo A. Hendriks[1], Michael Lund Nielsen[1], Hemmo Meyer[2], Julien P. Duxin[1], Guillermo Montoya[1], Niels Mailand[1,3,#]

[1]Protein Signaling Program, Novo Nordisk Foundation Center for Protein Research, University of Copenhagen, DK-2200 Copenhagen, Denmark; [2]Molecular Biology I, Faculty of Biology, University of Duisburg-Essen, 45117 Essen, Germany; [3]Center for Chromosome Stability, Department of Cellular and Molecular Medicine, University of Copenhagen, Blegdamsvej 3B, DK-2200 Copenhagen, Denmark

*Equal contribution; [#]Correspondence: niels.mailand@cpr.ku.dk

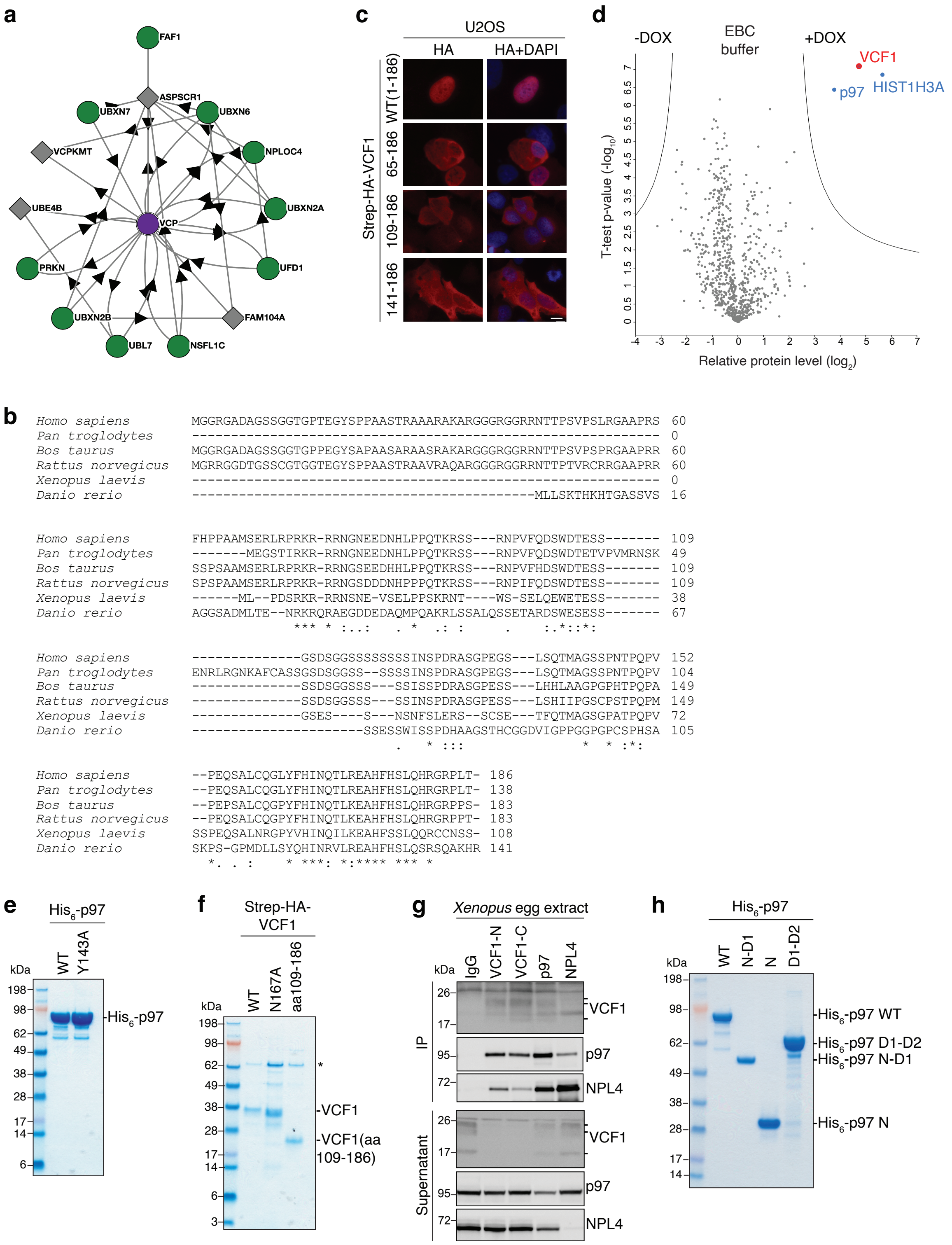

Supplementary Figure 1 (related to Figure 1).

VCF1 is a p97-interacting protein

a. BioPlex interactome (https://bioplex.hms.harvard.edu/) for p97 (VCP) in HEK293T cells [20].

b. Sequence alignment of selected vertebrate VCF1 orthologs, using ClustalOmega.

c. Representative images of U2OS cells transfected with indicated Strep-HA-VCF1 expression constructs and immunostained with HA antibody. Scale bar, 10 μM.

d. Mass spectrometry analysis of VCF1-interacting proteins. U2OS/GFP-VCF1 WT cells were treated or not with DOX for 16 h, subjected to GFP IP in non-denaturing EBC buffer and analyzed by mass spectrometry. Volcano plot shows enrichment of individual proteins (+DOX/-DOX ratio) plotted against the *P* value (**Supplementary Data 2**). Dashed lines indicate the significance thresholds (two-sided *t*-test, FDR<0.01, s_0=1).

e. Coomassie Blue staining of His_6-p97 proteins purified from *E. coli* and resolved by SDS-PAGE.

f. Coomassie Blue staining of Strep-HA-VCF1 proteins purified from HEK293-6E cells and resolved by SDS-PAGE.

g. Immunoblot analysis of *Xenopus* egg extracts subjected to IP with the indicated antibodies targeting the N-terminal (VCF1-N) or C-terminal (VCF1-C) region of VCF1, p97, NPL4 or an IgG control.

h. Coomassie Blue staining of full-length and truncated His_6-p97 proteins purified from *E. coli* and resolved by SDS-PAGE.

Data information: Data are representative of two (**g**) independent experiments with similar outcome.

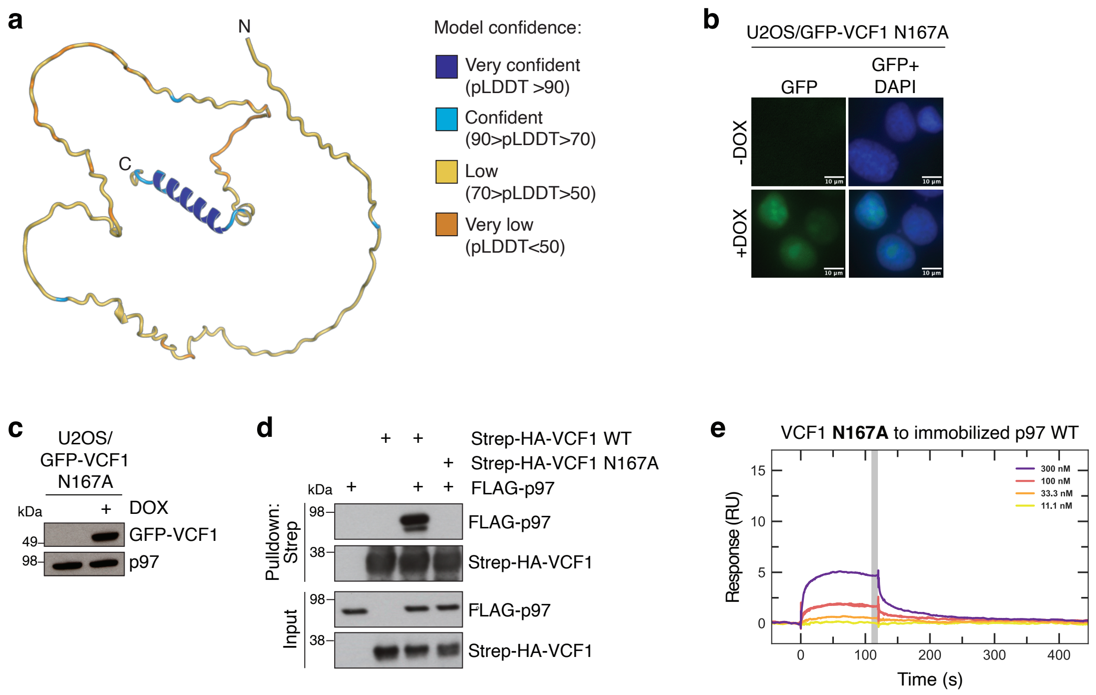

Supplementary Figure 2 (related to Figure 2).

VCF1 binds p97 via an α-helical motif (VRM)

a. AlphaFold2 model of human VCF1.

b. Representative images of U2OS/GFP-VCF1 N167A cells treated or not with Doxycycline (DOX) for 16 h. Scale bars, 10 μM.

c. Immunoblot analysis of U2OS/GFP-VCF1 WT cells treated as in (b).

d. Immunoblot analysis of *in vitro* binding reactions containing purified FLAG-p97 and Strep-HA-VCF1 proteins that were subjected to StrepTactin (Strep) pulldown.

e. SPR sensorgrams for the interaction between recombinant Strep-HA-VCF1 N167A and immobilized His_6-p97 WT. Grey bar denotes the region where the data was averaged to calculate the equilibrium binding responses at each concentration.

Data information: Data are representative of four (**c**) and three (**b**,**d**,**e**) independent experiments with similar outcome.

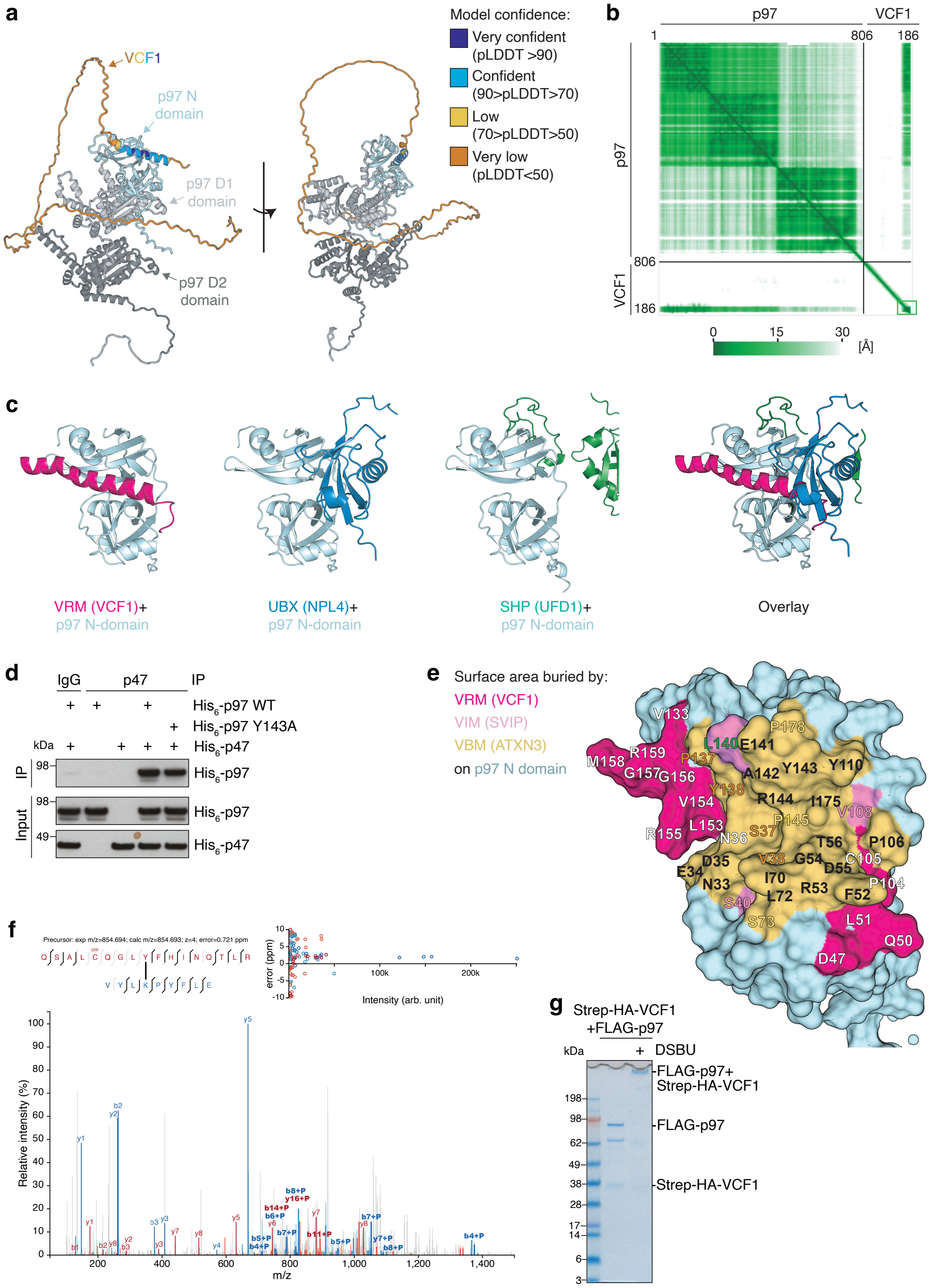

Supplementary Figure 3 (related to Figure 3).

Structural modeling of VCF1-p97 complex formation

a. AlphaFold-Multimer prediction of the complex between full-length VCF1 (color-coded by pLDDT value (measure of model confidence)) and a p97 monomer (colored by domains).
b. Predicted alignment error (PAE) plot for the VCF1-p97 complex. Green box indicates the VCF1 C-terminal helix.
c. Comparison of AlphaFold-Multimer models of the complexes formed between the p97 monomer (N-domain in pale cyan) and VCF1 (pink), NPL4 (blue) or UFD1 (green).
d. Immunoblot analysis of *in vitro* binding reactions containing purified His_6-p47 and His_6-p97 proteins that were subjected to IP using pre-immune serum (IgG) or p47 antibody.
e. Surface representation of the structure of the p97 N-domain (pale cyan), based on the p97-VCF1 AlphaFold-Multimer prediction in (a). Colored areas correspond to buried surface area calculated with PDBePISA (https://www.ebi.ac.uk/pdbe/pisa/) based on the p97-VCF1 (pink), p97-SVIP (rosa) and p97-ATXN3 (yellow) AlphaFold-Multimer models (**Figure 3c**). VCF1 covers a larger surface area on the p97 N-domain (~1119 Å^2) than SVIP (~758 Å^2) and ATXN3 (~739 Å^2). Residues predicted to reside at the p97-cofactor interface and used for the buried surface area calculation that are exclusively buried by VCF1, SVIP or ATXN3 are labeled in white, rosa and yellow, respectively. Residues common to the buried surface of all cofactor complexes are labeled in black, residues shared between VCF1 and ATX3 are labeled in orange, and residues shared between VCF1 and SVIP are labeled in green.
f. Fully annotated MS/MS spectrum demonstrating the DSBU crosslink between K136 in p97 and Y163 in VCF1. Top left; peptide fragment coverage map, with bold lines indicating identified fragments ('cm' indicates the presence of carbamidomethyl on the cysteine residue as a result of sample alkylation). Center; MS/MS spectrum with fragment ions originating from VCF1 in red, and fragment ions originating from p97 in blue ('+P' indicates fragment ions including the DSBU crosslink). Top right; fragment mass error in relation to fragment abundance in the MS/MS spectrum, with a maximum error tolerance of 10 ppm.
g. Coomassie Blue staining of purified FLAG-p97 and Strep-HA-VCF1 proteins incubated in the absence or presence of the crosslinker disuccinimidyl dibutyric urea (DSBU), demonstrating efficient DSBU-dependent crosslinking between FLAG-p97 hexamers and Strep-HA-VCF1.
Data information: Data are representative of two (**d**) independent experiments with similar outcome.

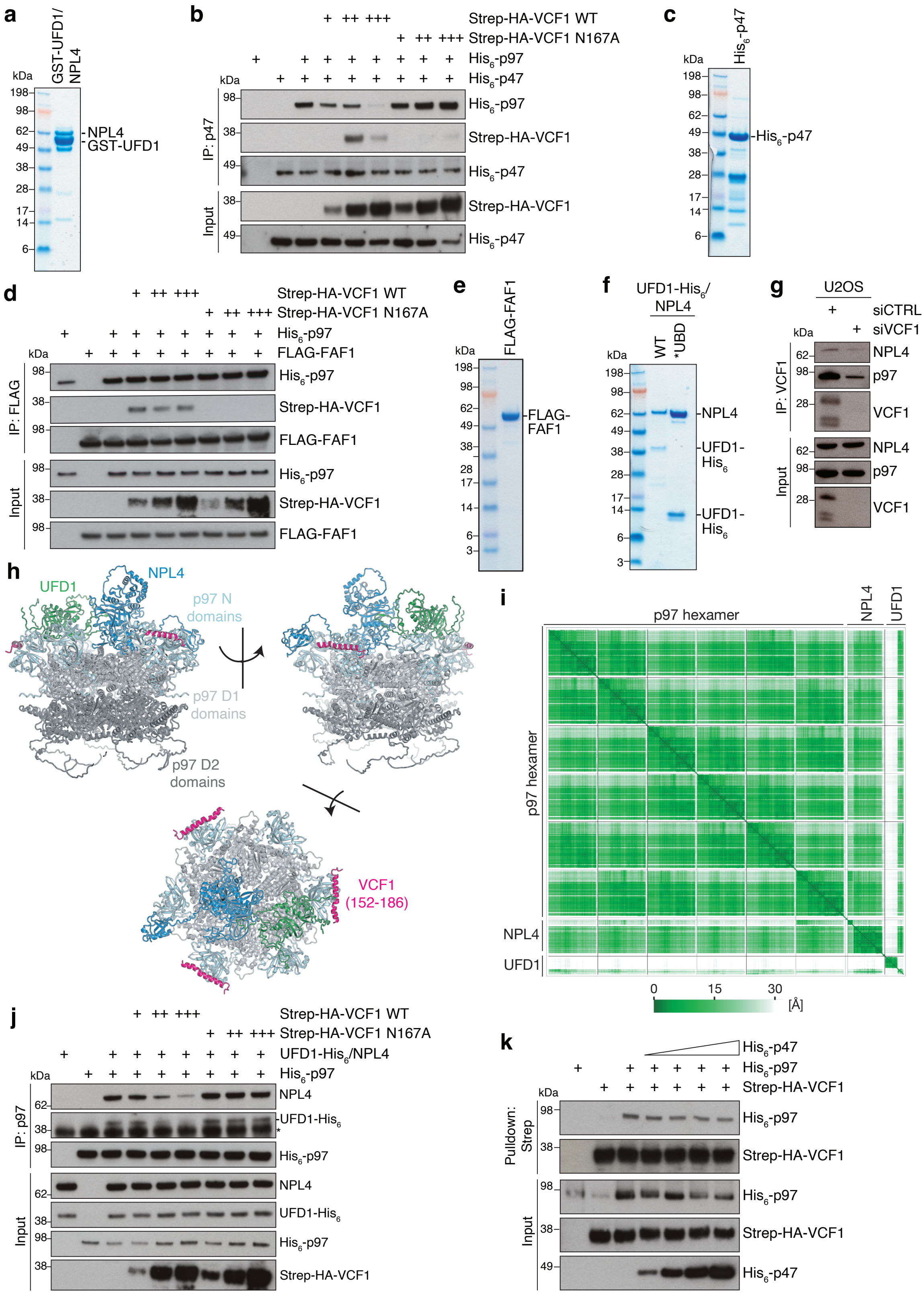

Supplementary Figure 4 (related to Figure 4).

VCF1 and other cofactors form joint complexes with p97

a. Coomassie Blue staining of GST-UFD1-NPL4 complex purified from *E. coli* and resolved by SDS-PAGE.

b. Binding reactions containing indicated combinations of purified Strep-HA-VCF1, His_6-p97 and His_6-p47 proteins were subjected to IP with p47 antibody and analyzed by immunoblotting.

c. Coomassie Blue staining of His_6-p47 purified from *E. coli* and resolved by SDS-PAGE.

d. Binding reactions containing indicated combinations of purified Strep-HA-VCF1, His_6-p97 and FLAG-FAF1 proteins were subjected to FLAG IP and analyzed by immunoblotting.

e. Coomassie Blue staining of FLAG-FAF1 purified from *E. coli* and resolved by SDS-PAGE.

f. Coomassie Blue staining of WT and ubiquitin binding-deficient (*UBD) UFD1-His_6-NPL4 complex purified from *E. coli* and resolved by SDS-PAGE.

g. Immunoblot analysis of U2OS cells transfected with indicated siRNAs and subjected to IP with VCF1 antibody.

h. Composite model of a potential human p97 hexamer bound to a UFD1-NPL4 heterodimer and three copies of the VRM helix, generated by superimposing three copies of the AlphaFold-Multimer prediction of monomeric p97-VRM (**Figure 3a**) onto three p97 subunits within an AlphaFold-Multimer model of the human p97-UFD1-NPL4 complex.

i. Predicted alignment error (PAE) plot for the p97-UFD1-NPL4 complex.

j. Binding reactions containing indicated combinations of purified Strep-HA-VCF1, His_6-p97 and UFD1-His_6-NPL4 proteins were subjected to IP with p97 antibody and analyzed by immunoblotting.

k. Binding reactions containing indicated combinations of purified Strep-HA-VCF1, His_6-p97 and increasing concentrations of His_6-p47 were subjected to StrepTactin (Strep) pulldown and analyzed by immunoblotting.

Data information: Data are representative of three (**j**) and two (**b**,**d**,**g**,**k**) independent experiments with similar outcome.

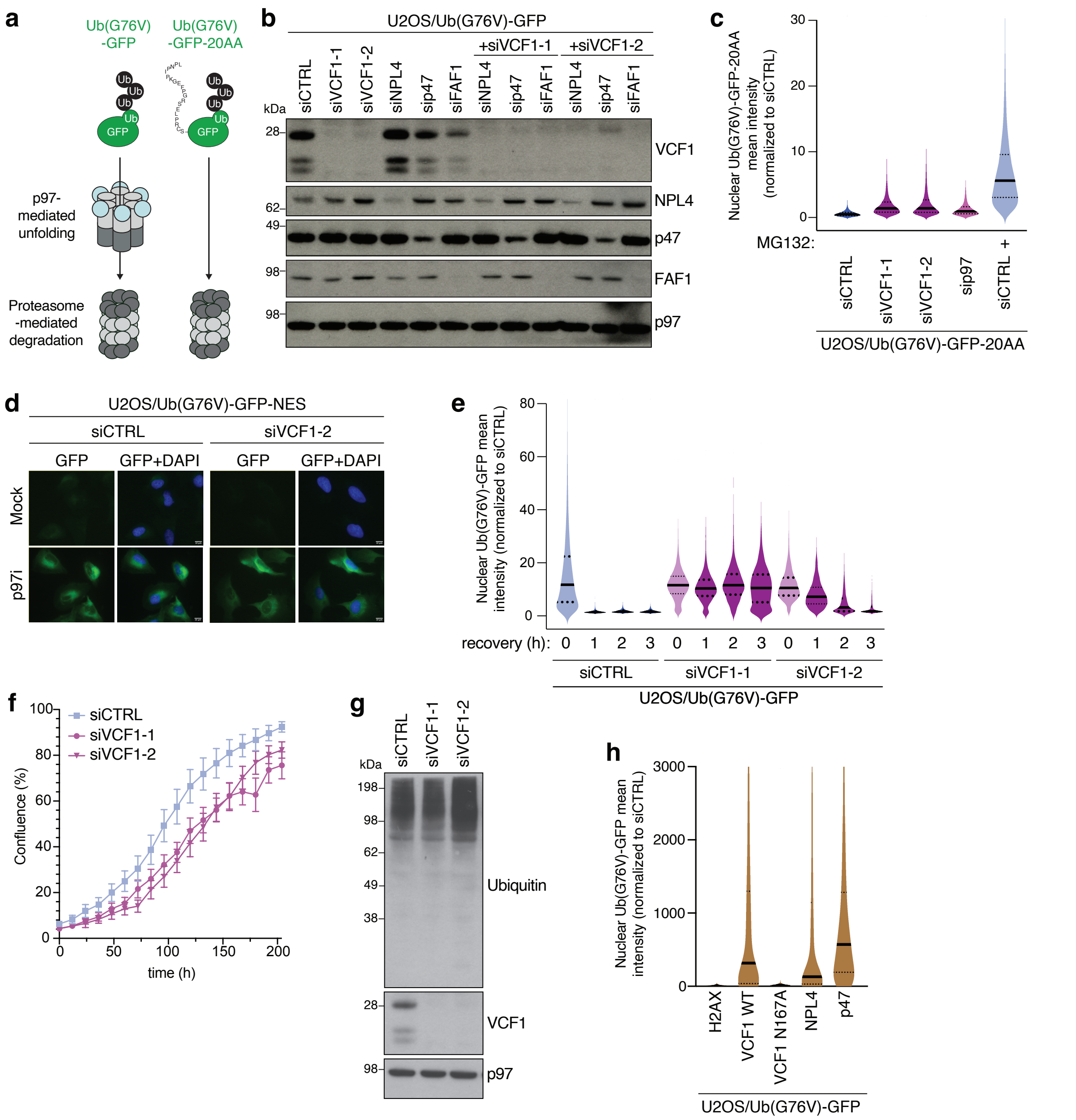

Supplementary Figure 5 (related to Figure 5).

VCF1 stimulates p97-UFD1-NPL4-dependent protein degradation in the nucleus

a. Schematic of p97- and/or proteasome-dependent degradation of fluorescent Ub(G76V)-GFP and Ub(G76V)-GFP-20AA reporters.

b. Immunoblot analysis of U2OS/Ub(G76V)-GFP cells in **Figure 5b** transfected with indicated siRNAs.

c. U2OS/Ub(G76V)-GFP-20AA cells were transfected with siRNAs and, where indicated, treated with the proteasome inhibitor MG132 for 2 h prior to fixation. Ub(G76V)-GFP-20AA intensity was analyzed by quantitative image-based cytometry QIBC (solid lines, median; dashed lines, quartiles; >10000 cells analyzed per condition).

d. Representative images of U2OS cells stably expressing Ub(G76V)-GFP-NES that were transfected with indicated siRNAs and treated or not with p97 inhibitor (p97i; NMS-873) for 2 h. Scale bars, 10 μM.

e. U2OS/Ub(G76V)-GFP cells transfected with siRNAs were treated with p97i for 4 h. Cells were then washed thoroughly and collected at the indicated times after p97i withdrawal. Ub(G76V)-GFP intensity was analyzed by QIBC (solid lines, median; dashed lines, quartiles; >10000 cells analyzed per condition).

f. Normalized logarithmic cell proliferation quantification for U2OS cells transfected with indicated siRNAs, determined by Incucyte image-based confluence analysis. Data from a representative experiment are shown.

g. Immunoblot analysis of U2OS cells transfected with indicated siRNAs.

h. U2OS/Ub(G76V)-GFP cells transfected with expression constructs encoding indicated FLAG-tagged plasmids were fixed and immunostained with FLAG antibody. Ub(G76V)-GFP intensity in FLAG-positive cells was analyzed by QIBC (solid lines, median; dashed lines, quartiles; >10000 cells analyzed per condition).

Data information: Data are representative of three (**d**,**e**,**g**,**h**) and two (**b**) independent experiments with similar outcome. Source data are provided as a Source Data file.

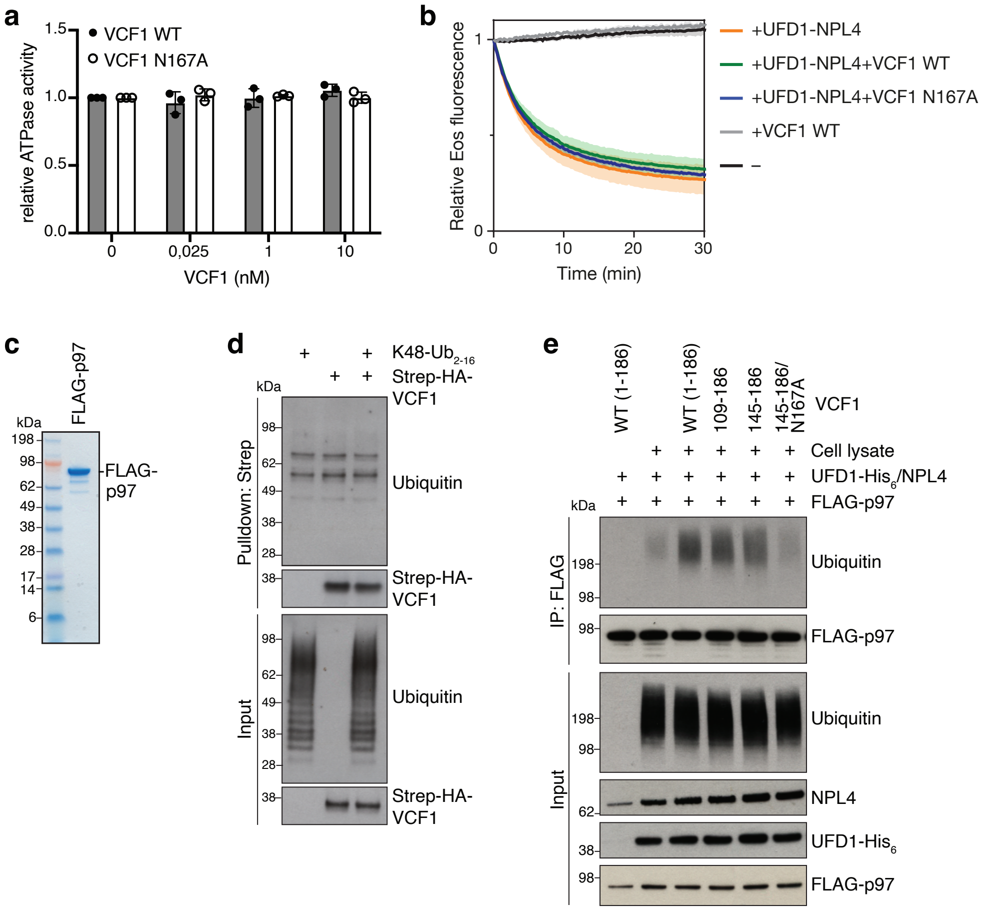

Supplementary Figure 6 (related to Figure 5).

VCF1 stimulates p97-UFD1-NPL4 interaction with ubiquitin conjugates

a. Relative ATPase activity of recombinant His$_6$-p97 protein (25 nM) incubated with indicated concentrations of purified Strep-HA-VCF1 proteins (*n*=3 independent experiments).

b. Unfolding of a recombinant poly-ubiquitinated mEos3.2 reporter protein (35 nM) by p97 (100 nM) with or without UFD1-NPL4 (500 nM), VCF1 WT (300 nM) and VCF1 N167A mutant (300 nM) as indicated. Loss of red mEos3.2 fluorescence was monitored by spectrometry (mean±SD; *n*=3 independent experiments (*n*=2 for VCF1 N167A)).

c. Coomassie Blue staining of FLAG-p97 purified from *E. coli* and resolved by SDS-PAGE.

d. *In vitro* binding reactions containing indicated combinations of purified Strep-HA-VCF1 and K48-linked ubiquitin chains (K48-Ub$_{2-16}$) were subjected to StrepTactin pulldown and analyzed by immunoblotting.

e. Immunoblot analysis of *in vitro* binding reactions containing indicated combinations of purified FLAG-p97, UFD1-His$_6$-NPL4 complex, whole cell extracts of U2OS cells and full-length or truncated VCF1 proteins that were subjected to FLAG IP.

Data information: Data are representative of three (d,e) independent experiments with similar outcome. Source data are provided as a Source Data file.

g

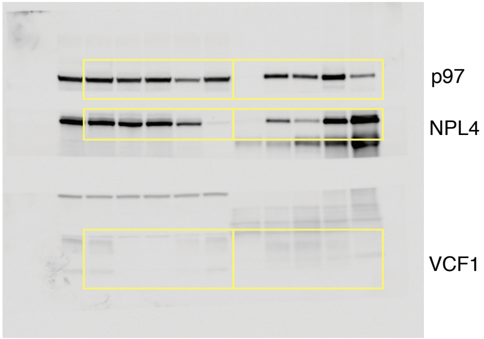

Uncropped scans for Supplementary Figure 1.

c

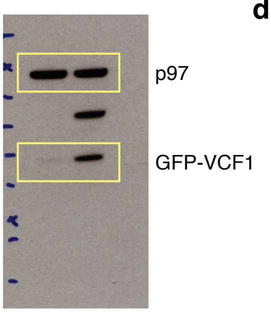

d

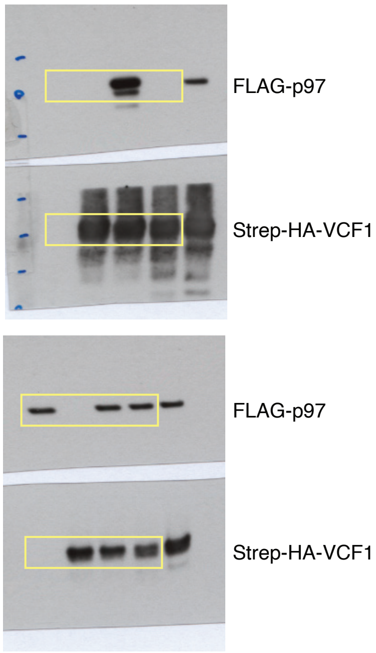

Uncropped scans for Supplementary Figure 2.

d

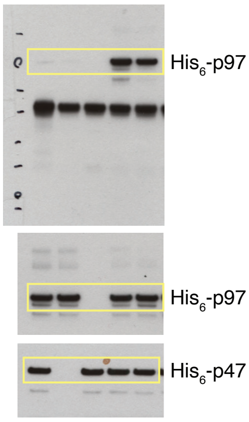

Uncropped scans for Supplementary Figure 3.

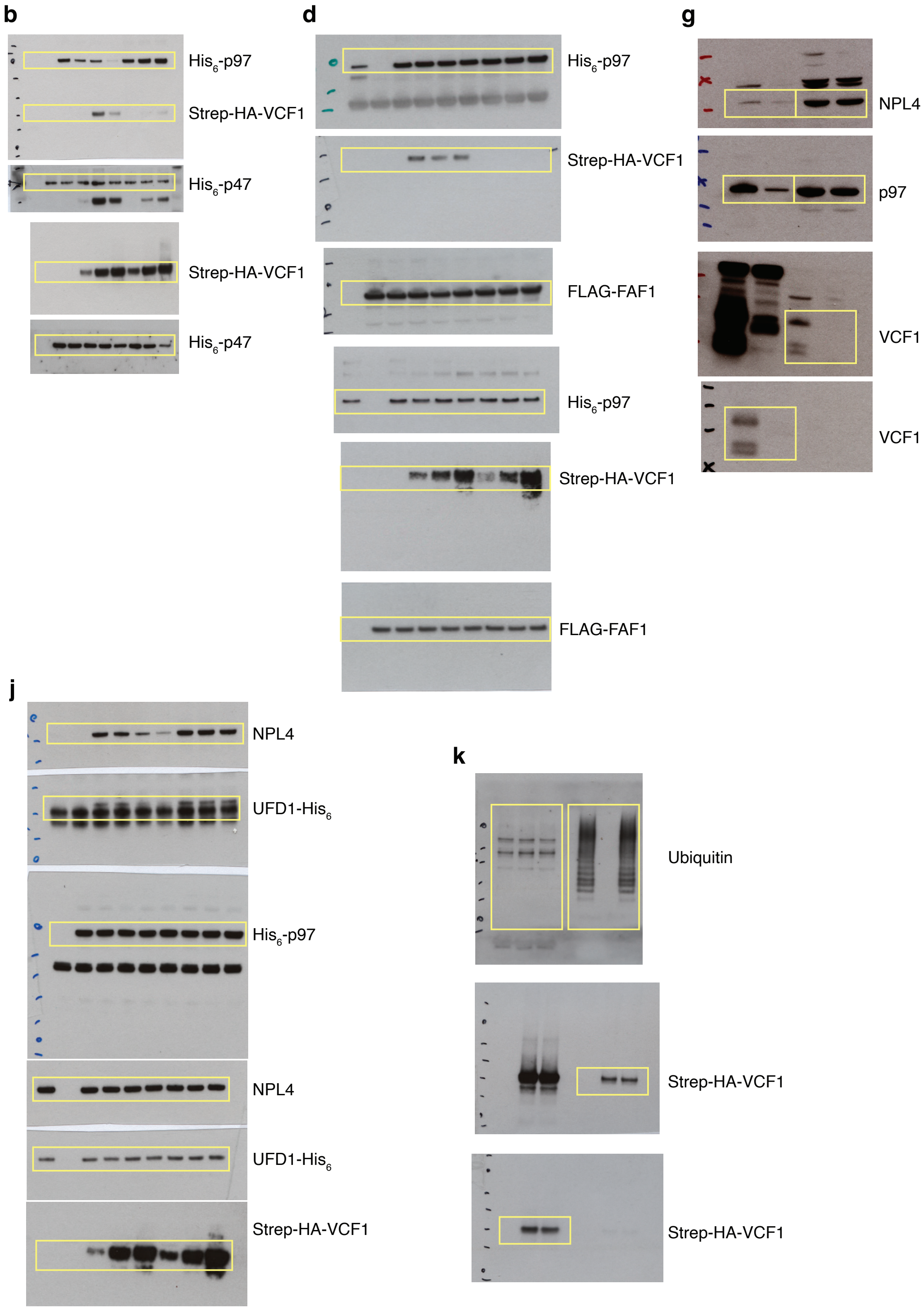

Uncropped scans for Supplementary Figure 4.

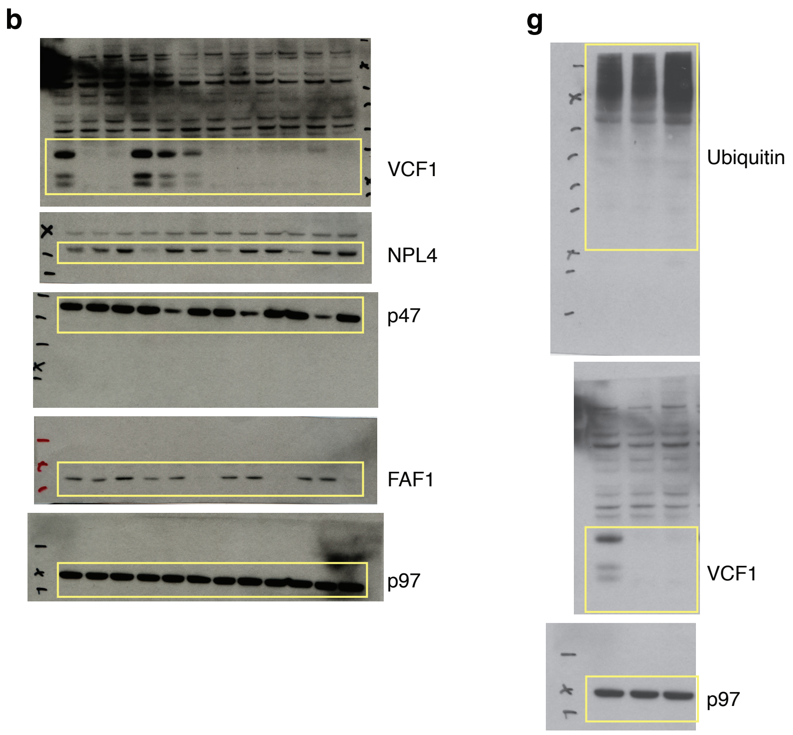

Uncropped scans for Supplementary Figure 5.

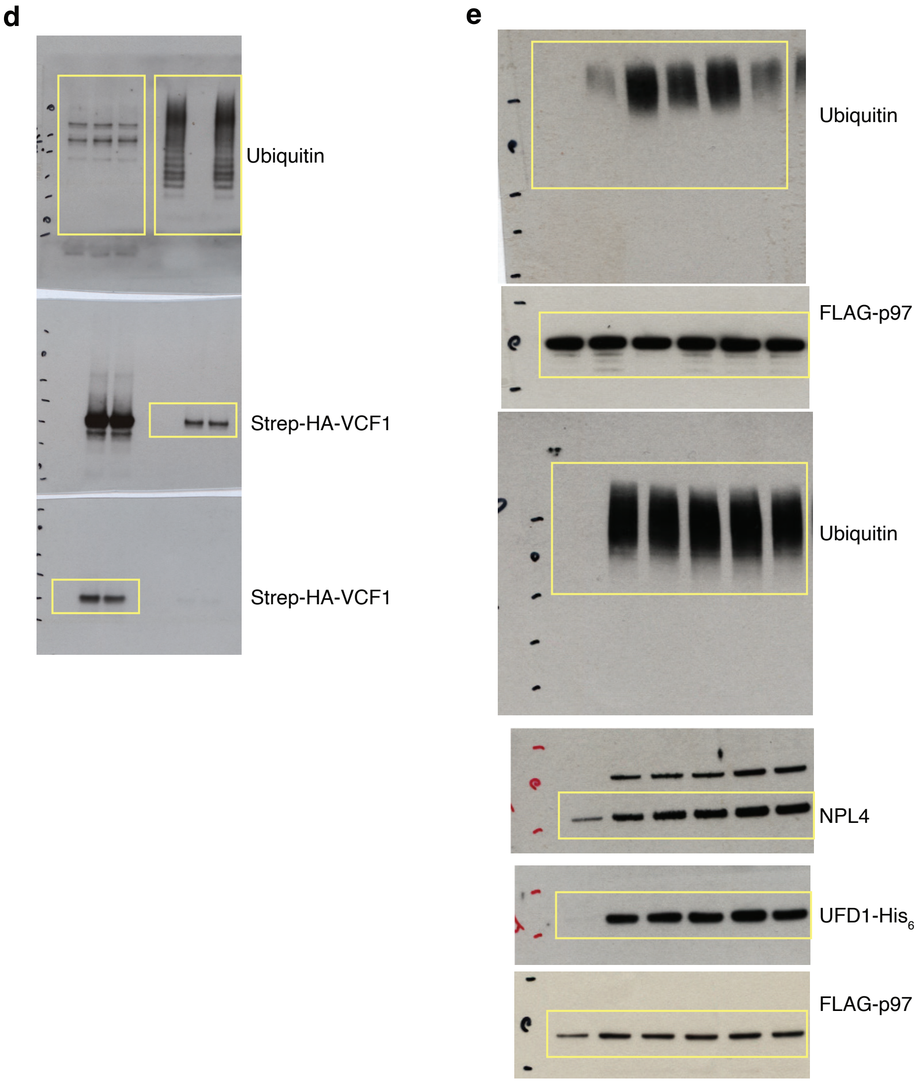

Uncropped scans for Supplementary Figure 6.
