# Supplementary material for: VCF1 is a p97/VCP cofactor promoting recognition of ubiquitylated p97-UFD1-NPL4 substrates
Source: Nat Commun. 2024 Mar 19;15:2459. doi: 10.1038/s41467-024-46760-4 (PMC10950897; doi:10.1038/s41467-024-46760-4)
Supplement: Supplementary file 3 — Description of Additional Supplementary Files [file 41467_2024_46760_MOESM3_ESM.pdf]

Description of Additional Supplementary Files

File name: Supplementary Data 1
Description: Mass spectrometry analysis of GFP-VCP1 WT IPs in partially denaturing RIPA buffer, related to Figure 1c.

File name: Supplementary Data 2
Description: Mass spectrometry analysis of GFP-VCP1 WT IPs in non-denaturing buffer, related to Supplementary Figure 1d.

File name: Supplementary Data 3
Description: Mass spectrometry analysis of GFP-VCP1 WT and N167A IPs in partially denaturing RIPA buffer, related to Figure 2e.
