# Supplementary material for: VCF1 is a p97/VCP cofactor promoting recognition of ubiquitylated p97-UFD1-NPL4 substrates
Source: Nat Commun. 2024 Mar 19;15:2459. doi: 10.1038/s41467-024-46760-4 (PMC10950897; doi:10.1038/s41467-024-46760-4)
Supplement: Supplementary file 7 — Reporting Summary [file 41467_2024_46760_MOESM7_ESM.pdf]

nature portfolio

Corresponding author(s): Niels Mailand

Last updated by author(s): Feb 23, 2024

Reporting Summary

Nature Portfolio wishes to improve the reproducibility of the work that we publish. This form provides structure for consistency and transparency in reporting. For further information on Nature Portfolio policies, see our Editorial Policies and the Editorial Policy Checklist.

Statistics

For all statistical analyses, confirm that the following items are present in the figure legend, table legend, main text, or Methods section.

n/a	Confirmed	
☐	☒	The exact sample size (*n*) for each experimental group/condition, given as a discrete number and unit of measurement
☐	☒	A statement on whether measurements were taken from distinct samples or whether the same sample was measured repeatedly
☐	☒	The statistical test(s) used AND whether they are one- or two-sided<br>*Only common tests should be described solely by name; describe more complex techniques in the Methods section.*
☒	☐	A description of all covariates tested
☐	☒	A description of any assumptions or corrections, such as tests of normality and adjustment for multiple comparisons
☐	☒	A full description of the statistical parameters including central tendency (e.g. means) or other basic estimates (e.g. regression coefficient) AND variation (e.g. standard deviation) or associated estimates of uncertainty (e.g. confidence intervals)
☐	☒	For null hypothesis testing, the test statistic (e.g. *F*, *t*, *r*) with confidence intervals, effect sizes, degrees of freedom and *P* value noted<br>*Give P values as exact values whenever suitable.*
☒	☐	For Bayesian analysis, information on the choice of priors and Markov chain Monte Carlo settings
☒	☐	For hierarchical and complex designs, identification of the appropriate level for tests and full reporting of outcomes
☒	☐	Estimates of effect sizes (e.g. Cohen's *d*, Pearson's *r*), indicating how they were calculated

Our web collection on statistics for biologists contains articles on many of the points above.

Software and code

Policy information about availability of computer code

Data collection	Incucyte S3 Live-Cell Analysis System (version 2021A), ScanR acquisition software (version 3.2.0; Olympus), AcquireMP (version 2022 R1).
Data analysis	ScanR analysis software (version 2.8.1; Olympus), Spotfire (version 10.5.0; Tibco), GraphPad Prism (version 9.5.1), MaxQuant (version 1.6.17.0), Perseus (versions 1.6.15.0), MeroX (version 2.0.1.4), AlphaFold-Multimer (version 2.3.1), PyMOL (version 1.2r3pre), BiaEvaluation (version 3.2.1), DiscoverMP (version 2022 R1).

For manuscripts utilizing custom algorithms or software that are central to the research but not yet described in published literature, software must be made available to editors and reviewers. We strongly encourage code deposition in a community repository (e.g. GitHub). See the Nature Portfolio guidelines for submitting code & software for further information.

Data

Policy information about availability of data

All manuscripts must include a data availability statement. This statement should provide the following information, where applicable:

- Accession codes, unique identifiers, or web links for publicly available datasets
- A description of any restrictions on data availability
- For clinical datasets or third party data, please ensure that the statement adheres to our policy

The mass spectrometry proteomics data generated in this study have been deposited to the ProteomeXchange Consortium 60 via the Proteomics Identifications (PRIDE) partner repository (http://www.ebi.ac.uk/pride) under dataset ID PXD043565 (https://proteomecentral.proteomexchange.org/cgi/GetDataset?

ID=PXD043565) (Supplementary Data 1-3) and dataset ID PXD043563 (https://proteomecentral.proteomexchange.org/cgi/GetDataset?ID=PXD043563) (Figure 3e). The AlphaFold models, results and input files generated in this study have been deposited to the Electronic Research Data Archive at University of Copenhagen (https://sid.erda.dk/share_redirect/h6SgLgmOGi). All other data supporting the findings of this study are available within the article and supplementary information. Source data are provided with this paper.

Research involving human participants, their data, or biological material

Policy information about studies with human participants or human data. See also policy information about sex, gender (identity/presentation), and sexual orientation and race, ethnicity and racism.

Reporting on sex and gender	N/A
Reporting on race, ethnicity, or other socially relevant groupings	N/A
Population characteristics	N/A
Recruitment	N/A
Ethics oversight	N/A

Note that full information on the approval of the study protocol must also be provided in the manuscript.

Field-specific reporting

Please select the one below that is the best fit for your research. If you are not sure, read the appropriate sections before making your selection.

☒ Life sciences ☐ Behavioural & social sciences ☐ Ecological, evolutionary & environmental sciences

For a reference copy of the document with all sections, see nature.com/documents/nr-reporting-summary-flat.pdf

Life sciences study design

All studies must disclose on these points even when the disclosure is negative.

Sample size	Sample sizes were based on previously reported data. No statistical method was used to predetermine sample size.
Data exclusions	No data were excluded from the analysis.
Replication	All experimental findings shown in this study were independently replicated at least twice with similar outcome. Information about replication is provided in the figure legends.
Randomization	The samples were not randomized. Randomization is generally not relevant for this study since we are working with cell populations and not test subjects.
Blinding	The investigators were not blinded to group allocation during data collection and analysis, but great care was taken to avoid bias.

Reporting for specific materials, systems and methods

We require information from authors about some types of materials, experimental systems and methods used in many studies. Here, indicate whether each material, system or method listed is relevant to your study. If you are not sure if a list item applies to your research, read the appropriate section before selecting a response.

Materials & experimental systems

n/a	Involved in the study	
☐	☒	Antibodies
☐	☒	Eukaryotic cell lines
☒	☐	Palaeontology and archaeology
☐	☒	Animals and other organisms
☒	☐	Clinical data
☒	☐	Dual use research of concern
☒	☐	Plants

Methods

n/a	Involved in the study	
☒	☐	ChIP-seq
☒	☐	Flow cytometry
☒	☐	MRI-based neuroimaging

Antibodies

Antibodies used	The following commercial antibodies were used: FLAG (A00187, GenScript, mouse, 1:1000 (RRID:AB_1720813)); HA (11867423001, Roche, rat, 1:1000 (RRID:AB_390918)); HA (sc-7392, Santa Cruz, mouse, 1:1000 (RRID:AB_627809)); HA (sc-805, Santa Cruz, rabbit, 1:1000 (RRID:AB_631618)); HA (MMS-101R, Covance, mouse, 1:1000 (RRID:AB_291262)); His6 (631212, Clontech, mouse, 1:500 (RRID:AB_2721905)); NPL4 (sc-365796, Santa Cruz, mouse, 1:500 (RRID:AB_10841920)); NPL4 (HPA021560, Atlas Antibodies, rabbit, 1:1000 (RRID:AB_1854586)); (UFD1 (sc-136114, Santa Cruz, mouse, 1:500 (RRID:AB_2213950)); UFD1 (611642, BD Biosciences, mouse, 1:1000 (RRID:AB_399070)); Ubiquitin (43124S, Cell Signaling Technology, rabbit, 1:1000 (RRID:AB_2799235)); Ubiquitin (sc-8017, Santa Cruz, mouse, 1:1000 (RRID:AB_628423)); p97 (MA3-004, Invitrogen, mouse, 1:5.000 (RRID:AB_2214638)); p97: Santa Cruz, mouse, sc-57492 (RRID:AB_793927)); GST (sc-138, Santa Cruz, mouse, 1:1000 (RRID:AB_627677)); p47 (NBP213677, Novus Biologicals, rabbit, 1:1000); FAF1 (Bethyl Laboratories, rabbit ((A302-810A), 1:1000). A sheep polyclonal VCF1 antibody was raised against full-length human VCF1 by MRC PPU Reagents and Services (University of Dundee, UK) and used at a 1:200 dilution. Antibodies against Xenopus p97, NPL4 and UFD1 44 were a kind gift from Olaf Stemmann (University of Bayreuth, Germany). The following antibodies against Xenopus proteins were raised against the indicated peptides (New England Peptide): VCF1 N-terminus (H2N-MLPDSRKRRRNSNEVSEC-amide), VCF1 C-terminus (Aoa-KEAHFSSLQQRCCNSS-OH). The VCF1 antibody used for immunoblotting was raised against full-length Xenopus laevis VCF1 (Biogenes). The VCF1 protein was tagged with His6 on the N-terminus and purified under denaturing conditions.
Validation	The specificity of antibodies against VCF1 (human and Xenopus), His6 (631212), HA (11867423001, sc-7392, sc-805), NPL4 (sc-365796), UFD1 (sc-136114), p97 (MA3-004), Ubiquitin (43124S, sc-8017), FLAG (A00187), p47 (NBP213677) and FAF1(A302-810A) were validated using appropriate negative controls included in this study (as shown in the figures). Other antibodies were used based on previous validation in the literature and/or manufacturer websites: HA (MMS-101R, Covance, mouse, 1:1000 (RRID:AB_291262)); NPL4 (HPA021560, Atlas Antibodies, rabbit, 1:1000 (RRID:AB_1854586)); UFD1 (611642, BD Biosciences, mouse, 1:1000 (RRID:AB_399070); p97: Santa Cruz, mouse, sc-57492 (RRID:AB_793927); GST (sc-138, Santa Cruz, mouse, 1:1000 (RRID:AB_627677)). The specificity of antibodies against Xenopus p97, NPL4 and UFD1 were validated using appropriate negative controls included in this study (as shown in the figures).

Eukaryotic cell lines

Policy information about cell lines and Sex and Gender in Research

Cell line source(s)	U2OS (female) cells were obtained from ATCC (catalog no. HTB-96). HEK293-6E (female) cells were a kind gift from Yves Durocher (National Research Council Canada, Montreal, Canada).
Authentication	The cell lines were not authenticated.
Mycoplasma contamination	All cell lines used in this study were regularly tested negative for mycoplasma infection.
Commonly misidentified lines (See ICLAC register)	Cell lines used in this study are not included in the ICLAC register of commonly misidentified cell lines.

Animals and other research organisms

Policy information about studies involving animals; ARRIVE guidelines recommended for reporting animal research, and Sex and Gender in Research

Laboratory animals	*For laboratory animals, report species, strain and age OR state that the study did not involve laboratory animals.*
Wild animals	*Provide details on animals observed in or captured in the field; report species and age where possible. Describe how animals were caught and transported and what happened to captive animals after the study (if killed, explain why and describe method; if released, say where and when) OR state that the study did not involve wild animals.*
Reporting on sex	*Indicate if findings apply to only one sex; describe whether sex was considered in study design, methods used for assigning sex. Provide data disaggregated for sex where this information has been collected in the source data as appropriate; provide overall numbers in this Reporting Summary. Please state if this information has not been collected. Report sex-based analyses where performed, justify reasons for lack of sex-based analysis.*
Field-collected samples	*For laboratory work with field-collected samples, describe all relevant parameters such as housing, maintenance, temperature, photoperiod and end-of-experiment protocol OR state that the study did not involve samples collected from the field.*
Ethics oversight	*Identify the organization(s) that approved or provided guidance on the study protocol, OR state that no ethical approval or guidance was required and explain why not.*

Note that full information on the approval of the study protocol must also be provided in the manuscript.
